# Partnering with law enforcement to deliver good public health: the experience of the HIV/AIDS Asia regional program

**DOI:** 10.1186/1477-7517-9-24

**Published:** 2012-07-09

**Authors:** Mukta Sharma, Anindya Chatterjee

**Affiliations:** 1 Technical Support Unit, HIV/AIDS Asia Regional Program, Bangkok, Thailand; 2 UNAIDS Regional Support Team, Johannesburg, South Africa

## Abstract

In the South-East Asia region, the drug control and supply reduction agenda is of high political importance. A multitude of law enforcement agencies are engaged in this work. Nationwide campaigns such as the “Strike- Hard” campaign in China or the “war on drugs” in Thailand dominate the landscape. Viet Nam’s response to drug use has historically focused on deterrence through punishment and supply-side measures. This policy environment is further complicated by lack of evidence-based drug dependence treatment in several settings. The public health consequences of this approach have been extremely serious, with some of the highest documented prevalence of preventable blood-borne viral infections, including HIV, and hepatitis B and C. The wider socioeconomic consequences of this have been borne by families, communities and the governments themselves.

The HIV/AIDS Asia Regional Program (HAARP) aims to stop the spread of HIV associated with drug use in South-East Asia and parts of southern China. HAARP works across five countries (Cambodia, China Burma, Laos, Viet Nam) chiefly through the Ministries of Health and Social Affairs, National Drug Control Agencies, and Public Security sectors, including prisons. HAARP has also engaged with UN agencies and a wide range of civil society organisations, including organisations of people who use drugs, to ensure their meaningful involvement in matters that directly affect them. We describe the experience of HAARP in implementing a large-scale harm reduction programme in the Sub-Mekong Region. HAARP chose to direct its efforts in three main areas: supporting an enabling environment for effective harm reduction policies, building core capacity among national health and law enforcement agencies, and supporting “universal access” goals by making effective, high-coverage services available to injecting drug users and their partners.

The activities supported by HAARP are humble yet important steps. However, a much higher political-level dialogue is needed. The current huge gap of human rights standards in drug control practices also needs to be bridged immediately. Public health that embraces a rights-based approach must be given its fair share of policy space, budget and influence.

## Background

The HIV/AIDS Asia Regional Program (HAARP) aims to stop the spread of HIV associated with drug use in South-East Asia and parts of southern China. HAARP is funded by the Australian Government (co-funded by the Government of the Netherlands in Viet Nam) and is one of the largest and longest-duration harm reduction programmes in Asia. In keeping with the commitments of the Australian Agency for International Development (AusAID) to the Paris and Accra Declarations, HAARP is implemented using a programme-based approach. HAARP works across five countries (Cambodia, China Burma, Laos, Viet Nam) chiefly through the Ministries of Health and Social Affairs, National Drug Control Agencies, and Public Security sectors, including prisons. HAARP has also engaged with UN agencies and a wide range of civil society organisations, including organisations of people who use drugs, to ensure their meaningful involvement in matters that directly affect them.

In the South-East Asia region, the drug control and supply reduction agenda is of particularly high political importance. Nationwide campaigns such as the “Strike- Hard” campaign in China (*Yanda*) or the “war on drugs” in Thailand (*Songkram Yaseptid*) dominate the landscape [1]. Viet Nam’s response to drug use has historically focused on deterrence through punishment and supply-side measures [2]. A multitude of law enforcement agencies are engaged in this work, ranging from border security forces, the army, police and specialised narcotics control forces. Drug control agencies in the region, often headed by Deputy Prime Ministers (e.g. in Cambodia and Viet Nam), wield significantly more influence than health or social welfare agencies.

Effective HIV prevention and AIDS care requires the use of humane and evidence-based harm reduction policies, which are poorly supported or understood by the law enforcement sector. A particular feature of the South-East Asian region is that countries currently pursue both goals – one (drug control) seen as more important than the other (public health). This policy environment is further complicated by lack of evidence-based drug dependence treatment in several settings. Quackery, boot camps, labour camps and use of forced treatment are common responses. This kind of compulsory treatment and incarceration is in contravention of international human rights conventions [3].

The public health consequences of this approach have been extremely serious, with some of the highest documented prevalence of preventable blood-borne viral infections, including HIV, and hepatitis B and C [4]. People who use drugs have disproportionately high rates of tuberculosis, overdose, suicide and trauma [4]. The wider socioeconomic consequences of this have been borne by families, communities and eventually by the governments themselves – with overloaded prison systems, human rights-related abuse and consequences of such law enforcement campaigns. For example, between 1996 and 2002, the Thai prison population increased by 250%; 53% of all Thai prisoners were incarcerated for drug-related offences (70% in Bangkok) [5]. In Viet Nam, the number of government-funded compulsory drug detention centres increased by over 60%, from 80 in 1995 to 129 by June 2010 [6]. The reported number of (registered) drug users in Viet Nam in June 2011 was 149,900 [7].

We describe the experience of HAARP in implementing a large-scale harm reduction programme in the Sub-Mekong Region. Effective engagement and collaboration with the drug control and law enforcement sectors was critical to the ability of the Program to provide and expand harm reduction services to people who use drugs within a restricted policy space. We describe the strategies and activities that the Program used across the five countries, using the current literature and data collected as part of routine monitoring and evaluation in HAARP to support our assertions. We discuss how law enforcement actors can be positively influenced by other agents, including civil society, in order to support effective public health actions.

## Strategies used by HAARP

Delivering effective and large-scale harm reduction programmes within such a challenging and contradictory policy environment needed careful planning and strategic thinking. This involved consideration of the nature of partnerships needed, as well as the influence and capacity of concerned institutions in supporting a scaled-up response to the epidemic of blood-borne viral infections.

The need to work at multiple levels and partners was identified early. HAARP chose to direct its efforts in three main areas: supporting an enabling environment for effective harm reduction policies, building core capacity among national health and law enforcement agencies, and supporting “universal access” goals by making effective, high-coverage services available to injecting drug users (IDUs) and their partners.

### Supporting an enabling environment

A crucial decision was to position the Program in such a way that regular policy dialogue was possible between drug control, social affairs and health sectors. At the operational level, it was critical to involve drug control, social welfare and AIDS control agencies to ensure collaboration and efficiency (Table 1). Project Steering Committees were set up at the national and provincial levels, co-chaired by government officials from law enforcement (e.g. Ministry of Public Security), Ministry of Social Affairs and the health sector (e.g. Ministry of Health, Centers for Disease Control, national AIDS control agencies).

**Table 1:** Strategies for engaging with law enforcement agencies by the HIV/AIDS Asia Regional Program (HAARP)

**No.**	**Strategies**	**Countries**
1.	Locating the programme in drug control agencies and signing agreements of partnership	Cambodia, Laos, Myanmar
2.	Working with existing national inter-agency mechanisms, e.g. national Task Forces	Cambodia, Laos
3.	Involving law enforcement in the governance of the programme	All HAARP countries
4.	Supporting large-scale training of law enforcement officials on harm reduction	Myanmar, China, Cambodia, Viet Nam
5.	Providing financial and technical resources to develop capacity of drug control agencies	Cambodia and Viet Nam
6.	Supporting HIV-related activities in prisons and drug detention centres	Viet Nam and China
7.	Supporting study trips of senior officials and politicians	Cambodia, Viet Nam, Laos, China

**Table 2:** Volume of harm reduction training for law enforcement staff in community and closed settings during 2009–2011

**Trainee type**	**2009**	**2010**	**2011**	**TOTAL**
Person times law enforcement staff at training academies and in the community	2119	2217	1525	**5861**

The engagement with law enforcement partners was well resourced, with budgets allocated to specific agencies in some cases to strengthen their capacity and coordination ability to respond to HIV, AIDS and treatment of substance use. This was also done in partnership with UN agencies in two countries (United Nations Office on Drugs and Crime and the World Health Organization).

HAARP also engages in active advocacy with law enforcement agencies at the community, provincial and national levels to address informal and formal policies and practices that either prevented the introduction (as in Laos) or hampered the scale-up of harm reduction service delivery. Leveraging the bilateral relationships between Australia and HAARP countries, and engaging senior-level officials, politicians and sometimes Deputy Prime Ministers in policy dialogue on evidence-based and humane approaches to drug use and HIV was found to be handy.

### Building core capacity

A key thrust has been to help law enforcement personnel understand the need for policy coherence on drug and HIV control, and provide them with the knowledge and skills to support harm reduction programmes (see Table 2). In order to build the capacity of law enforcement personnel, HAARP partnered with law enforcement training institutions. For example, the Yunnan Police Training Academy, with financial support from HAARP Yunnan, has been a key training agency within China and for other countries including Cambodia, Myanmar, and Laos.

A harm reduction training curriculum was also developed, which was contextualised and translated to suit the requirements of different HAARP country programmes. In some cases (e.g. Cambodia), this curriculum has been approved to be part of the national police training curriculum. In Myanmar, this curriculum forms the basis of a Drug and HIV/AIDS Awareness Training for Police Officers at the Central Police Academy (*Zee Pin Gyi*). Pre- and post-test training questionnaires show a significant improvement in HIV- and drug-related knowledge. HAARP has also provided 6849 person-times training in closed settings.

#### Helping law enforcement to come on board to support harm reduction service delivery in Laos

In 2010, rapid assessment surveys undertaken by HAARP confirmed the existence of worrying levels of HIV prevalence among IDUs in four districts of Lao PDR bordering Viet Nam, in Phongsaly and Houaphan provinces; nearly 17% of them were living with HIV infection.^a^ These findings led to a national-level discussion convened by the National Task Force on HIV and Injecting Drug Use. This body is co-chaired by the Lao Commission on Drug Control (LCDC) and the Ministry of Health, and has representatives from the Ministry of Public Security (MPS), UN organisations and civil society groups. While international best practice indicated that Lao should implement high-coverage harm reduction interventions as early as possible, Government understanding and support for such an initiative was mixed, with health sector representatives being fully supportive, and LCDC and MPS having some reservations. Not only was harm reduction a very new concept in Lao PDR, but it also posed policy contradictions for law enforcement agencies who have worked hard to control supply and demand. There were also concerns regarding the feasibility of delivering harm reduction interventions in geographically remote and sparsely populated locations using the standard service modalities, and community acceptance of harm reduction approaches in rural societies.

There was clearly a need for more first-hand information, understanding and reassurance that harm reduction could be culturally and politically appropriate in the Lao context before these agencies could be asked to support enabling policies and effective public health action. This information and reassurance needed to be based on examples not from developed-country settings, but from resource-poor, culturally and politically similar locations.

After consultation between government partners, UNODC and HAARP, it was agreed that key government counterparts would be funded through HAARP to attend a study visit to Viet Nam – a country with which Laos PDR has traditionally had very close links.

The Chairman of the Lao National Commission for Drug Control and Supervision, the Vice Minister of the Ministry of Public Security, the Vice minister of the Ministry of Health, the Deputy head of the Centre for HIV and Sexually Transmitted Infections (CHAS), the Co-Chair of National Task Force, and the UNODC Representative joined a nine-member delegation on a visit to Hanoi and Haiphong from 21 to 24 August 2011. For the first time, both policy-level decision-makers and technical experts from the Lao PDR jointly conducted a study visit of this kind.

The objective was to study how Viet Nam had addressed the issue of injecting drug use, HIV and AIDS, and how policy contradictions had been managed and minimised to support service provision.

**Figure 1:**
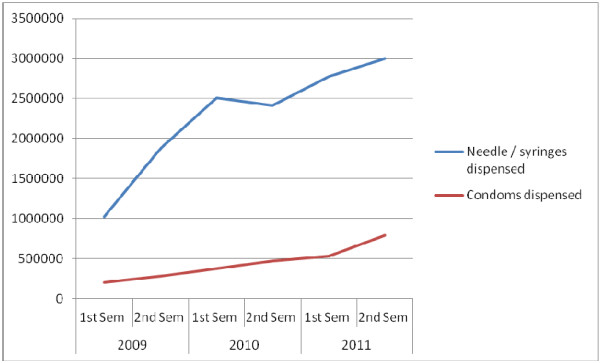
**Photo: Ministers and delegation members from Laos PDR watch methadone dispensing for opioid-dependent individuals in Haiphong.***Courtesy:* UNODC Lao PDR.

The participants met and discussed with high-ranking health and public security officials at the national and provincial levels, as well as drug users and health staff on the issue of drug control, drug use, HIV/AIDS, and harm reduction. They observed the flexibility and coordination between the law enforcement and health sectors to provide better opportunities for IDUs to access HIV treatment, care, support and prevention, specifically regarding opioid substitution therapy and clean needle and syringe programmes (*see* Figure 1).

After the visit to Laos, high-level policy-makers agreed to pilot similar programmes in two provinces of Houaphan and Phongsaly in northern Laos, although the specificities of service delivery modalities and terminology used to describe clean needle–syringe programmes have been adjusted to fit with the realities on the ground in these provinces. Plans to commence service delivery are currently being worked out.

#### Supporting drug users’ organisations to engage law enforcement for improving policy

The National Ministry of Public Security in China had set up a Dynamic Surveillance System (DSS) focusing on people who had a criminal record in 2006. Sixty-eight thousand former drug users had been included in the DSS till 2010. The DSS allowed data entry but no exit; with Public Security Departments at local levels not being entitled to modify the information in the DSS. It influenced the daily life of 68,000 former drug users, as policemen would interrogate them and undertake urine testing when these individuals used their identity cards in different situations such as renting an apartment or attempting to secure a place at school for their children. The Yunnan Harm Reduction Network staff observed that registered former drug users were facing serious difficulties due to the impact of this system on their daily lives. HAARP supported the Yunnan Drug User Network in collecting evidence of the impact of this policy – supporting the design of the research, data collection tool development, and help with data analysis, report writing, organization and participation in advocacy activities.

The research focused on former drug users who had been abstinent or on methadone maintenance therapy (MMT) for 3–5 years, and included their family members, community members, and local policemen. Information was collected via a survey (*N* = 200) and 17 in-depth interviews in two municipalities and seven counties in Yunnan and Guangxi Provinces.

The results of the research indicated that former drug users were harassed when checking into hotels (92.9%), applying for papers and documents (88.4%), travelling (95.9%), and renting apartments (14.5%). The DSS negatively influenced their work, life, family, marriage, and mental health, and continued to stigmatize them. It also negatively impacted the motivation of thousands of drug users who had been on MMT for years but continued to be treated like offenders. The research highlighted the vast expense and time expended by law enforcement personnel in keeping their obligations of implementing the DSS.

A joint advocacy effort to reverse this policy was undertaken in partnership with eight non-governmental partners. The results were presented in different venues and forums at the provincial and national levels, and shared via key messengers with the Yunnan Narcotics Bureau, the National Narcotic Control Office, as well as the media. A decision was taken in December 2010 to change the national regulation on Narcotics Control and 68,000 former drug users were successfully removed from the DSS. On 22 June 2011, the new *Narcotics Control Law* was adopted. Article 7 clearly states that the former drug users who had been abstinent or on MMT for three years and above were no longer under supervision as part of the DSS.

### Providing effective, high-coverage services

#### Achieving scale-up

**Figure 2:**
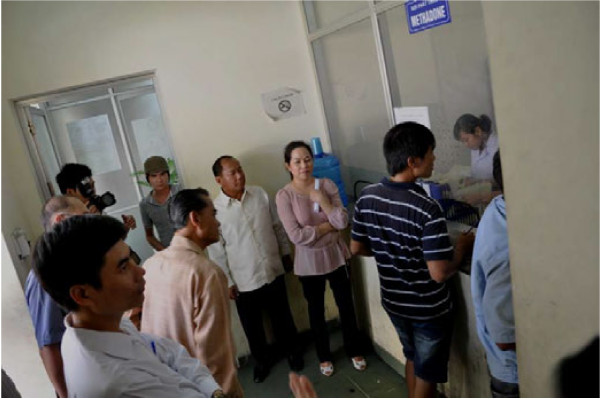
Needle/syringes and condoms dispensed through HAARP service sites between January 2009 and December 2011.

Since the beginning of 2009, a total of 13,592,475 needle/syringes and 2,651,492 condoms have been dispensed through HAARP country programmes. There has been a 300% scale up in terms of individual drug users reached and, despite the difficult policy environment, the total number of needle/syringe programme sites has increased from 11 in 2008 to 82 in 2012. In the second half of 2011 alone, HAARP reached 21,606 individuals (17,417 IDUs and 4189 non-injecting drug users) with harm reduction services. HAARP Cambodia saw the setting up of the first MMT facility located within the Soviet–Khmer Hospital in Phnom Penh in July 2010. The National Authority for Combating Drugs (NACD) as well as the Deputy Prime Minister’s office have been very supportive of this initiative. The intervention has been recently evaluated as being effective and plans are in place to scale up MMT across other sites in Cambodia (Figure 2).

A significant part of this scale up can be attributed to more effective intersectoral dialogue and common understanding of what the issues are at the ground level, e.g. law enforcement practices that disrupt services, agreement on not arresting outreach workers, and treating drop-in centres as safe places for people who use drugs.

## Challenges

The lack of coherence across drug and AIDS policy remains an unfinished agenda in the region. While service delivery has been scaled up in some countries through collaboration such as with HAARP and, more importantly, where national leadership supported such approaches, the thrust of drug policies in the direction of a “drug-free” outcome has continued to stand in the way of real progress. Policy stalemates have negatively impacted programme implementation in some settings, notably in Cambodia. The intensification of law enforcement campaigns against people who use drugs as part of the Commune Competitive Plan which became operational in 2008, and the Village/Commune Safety Policy enacted in August 2010, have been in direct conflict with Cambodia’s National AIDS Strategic Plan. No new NSP licenses were issued by the NACD since 2005. This has limited the number of agencies that can implement such programmes in the community and thereby resulted in a lack of expansion of such services.

## Future steps

A much higher political-level dialogue than is now the case is the need of the hour to innovatively explore the costs and consequences of different policy choices. The current huge gap of human rights standards in drug control practices also needs to be bridged immediately. Public health that embraces a rights-based approach has to claim its fair share of policy space, budget and influence, which is long overdue. The activities supported by HAARP are humble yet important steps in that direction.

## Endnotes

^a^It should be noted that this prevalence was found in a small sample (*N* = 49) and should not be seen as an estimate of national-level prevalence among IDUs in Lao PDR.
